# Neutrophil extracellular trap-associated protein in cerebrospinal fluid for prognosis evaluation of adult bacterial meningitis: a retrospective case-control study

**DOI:** 10.1186/s12879-024-09423-9

**Published:** 2024-05-27

**Authors:** Song Han, Suge Yang, Zhongzheng Chang, Yun Wang

**Affiliations:** 1 The Second Hospital of Shandong University, Cheeloo College of Medicine,Shandong University, Jinan, 250033 China; 2 Department of Neurology?Qilu Hospital (Qingdao), Cheeloo College of Medicine, Shandong University, Qingdao, 266035 China

**Keywords:** Bacterial meningitis, Neutrophil extracellular traps, Myeloperoxidase, Biomarkers

## Abstract

**Background and objectives:**

Central nervous system infections, typified by bacterial meningitis, stand as pivotal emergencies recurrently confronted by neurologists. Timely and precise diagnosis constitutes the cornerstone for efficacious intervention. The present study endeavors to scrutinize the influence of inflammatory protein levels associated with neutrophils in cerebrospinal fluid on the prognosis of central nervous system infectious maladies.

**Methods:**

This retrospective case series study was undertaken at the Neurology Department of the Second Hospital of Shandong University, encompassing patients diagnosed with infectious encephalitis as confirmed by PCR testing and other diagnostic modalities spanning from January 2018 to January 2024. The quantification of MPO and pertinent inflammatory proteins within patients’ cerebrospinal fluid was accomplished through the utilization of ELISA.

**Results:**

We enlisted 25 patients diagnosed with bacterial meningitis, ascertained through PCR testing, and stratified them into two groups: those with favorable prognoses (*n* = 25) and those with unfavorable prognoses (*n* = 25). Following assessments for normality and variance, notable disparities in CSF-MPO concentrations emerged between the prognostic categories of bacterial meningitis patients (*P* < 0.0001). Additionally, scrutiny of demographic data in both favorable and unfavorable prognosis groups unveiled distinctions in CSF-IL-1β, CSF-IL-6, CSF-IL-8, CSF-IL-18, CSF-TNF-α levels, with correlation analyses revealing robust associations with MPO. ROC curve analyses delineated that when CSF-MPO ≥ 16.57 ng/mL, there exists an 83% likelihood of an adverse prognosis for bacterial meningitis. Similarly, when CSF-IL-1β, CSF-IL-6, CSF-IL-8, CSF-IL-18, and CSF-TNF-α levels attain 3.83pg/mL, 123.92pg/mL, 4230.62pg/mL, 35.55pg/mL, and 35.19pg/mL, respectively, there exists an 83% probability of an unfavorable prognosis for bacterial meningitis.

**Conclusion:**

The detection of neutrophil extracellular traps MPO and associated inflammatory protein levels in cerebrospinal fluid samples holds promise in prognosticating bacterial meningitis, thereby assuming paramount significance in the prognostic evaluation of patients afflicted with this condition.

## Introduction

Bacterial Meningitis (BM) manifests as an acute inflammatory reaction affecting the arachnoid, pia mater, cerebrospinal fluid, and ventricles, provoked by pyogenic bacteria. It typically entails mild involvement of the brain and spinal cord surfaces, often coinciding with purulent encephalitis or brain abscess formation. Notably, the advent of vaccines targeting pneumococcal pneumonia, meningococcal meningitis, and Haemophilus influenzae type b in the United States has enabled infection prevention, symptom alleviation, and prognosis enhancement through vaccination [1–3]. Over the past two decades, China has witnessed a gradual reduction in the prevalence of bacterial meningitis, substantially lowering childhood mortality rates attributable to meningitis-related illnesses. However, among adults, waning immune function with age leads to a decline in vaccine-induced antibody levels, rendering bacterial meningitis and viral encephalitis significant concerns. These maladies not only inflict suffering and discomfort upon afflicted individuals but also impose substantial burdens on families and society at large. To combat this challenge, the World Health Organization (WHO) has devised a roadmap aimed at eradicating meningitis by 2030 [4]. Hence, timely identification and intervention in cases of meningitis hold the potential to ameliorate patient prognoses and mitigate complication occurrences.

Central nervous system infections such as bacterial meningitis represent a challenging emergency often faced by neurologists, with timely and accurate diagnosis being the prerequisite for effective treatment. Medical history and physical examination can provide important diagnostic clues, while cerebrospinal fluid analysis, neuroimaging, and electroencephalogram are necessary auxiliary means and decision-making bases. The definitive diagnosis of etiology mainly depends on cerebrospinal fluid microbiological staining, culture, and identification; detection of specific antibodies (IgM) in blood and cerebrospinal fluid; PCR testing of pathogen nucleic acids in cerebrospinal fluid; and brain biopsy pathology. However, the diagnosis of the etiology of central nervous system infections still faces many difficulties in terms of accuracy and timeliness, requiring high diagnostic technical requirements, with around half of encephalitis cases in clinical practice still unable to determine the cause despite comprehensive examinations.

Neutrophils are the most abundant white blood cells in the human innate immune system, also known as the nonspecific immune system. They represent the first line of cellular defense against invading pathogens in circulation. Neutrophil Extracellular Traps (NETs) are a meshwork structure released extracellularly by activated neutrophils, with free deoxyribonucleic acid (cfDNA) as the backbone, embedded with proteins such as histones, neutrophil elastase, myeloperoxidase (MPO), and proteinase G. Since 2004, NETs have gradually become a hot research topic in infectious and immune-related diseases. Additionally, certain cytokines play important roles in the inflammatory response during the course of BM, such as tumor necrosis factor and interleukin-1, which can stimulate endothelial cell adhesion and promote the entry of neutrophils into the central nervous system, triggering the inflammatory process. However, investigations into NETs-related protein content in cerebrospinal fluid during disease states remain scant. Consequently, this study endeavors to elucidate the prognostic utility of neutrophil extracellular trap-associated proteins in cerebrospinal fluid among adults afflicted with bacterial meningitis.

## Methods

### Research subjects

We conducted a retrospective study from January 2018 to January 2023 at the Department of Neurology, Second Hospital of Shandong University, involving patients suspected of having central nervous system infectious diseases. Patient medical records related to lumbar puncture were collected and reviewed. Based on clinical guidelines and relevant literatuer [5–7].we selected bacterial meningitis patients (*n* = 25) according to inclusion criteria and excluded those who did not meet the criteria.

This research involving human participants was reviewed and approved by the Ethics Committee of the Second Hospital of Shandong University (Approval No: KYLL-2023LW034). Written informed consent was obtained from the patients/participants for their participation in this study.

### Inclusion criteria

Patients diagnosed with bacterial meningitis (BM group, *n* = 25) demonstrated clinical presentations and laboratory parameters consistent with the diagnostic criteria for bacterial meningitis delineated in the 2016 edition of the “European Society of Clinical Microbiology and Infectious Diseases Acute Bacterial Meningitis Diagnosis and Treatment Guidelines.” [8].

①Clinical Presentation: Acute onset accompanied by fever, headache, vomiting, signs of meningeal irritation on clinical examination, increased intracranial pressure, and significant leukocytosis.

②Laboratory Results:

(a) Blood routine examination showed increased white blood cell count, typically ranging from (10–30)x10^9/L, predominantly neutrophils, occasionally normal or above 40 × 10^9/L.

(b) Cerebrospinal fluid examination revealed elevated pressure, turbid or purulent appearance, significantly increased cell count, predominantly neutrophils, usually ranging from (1000–10,000)x10^6/L. Elevated protein levels, decreased glucose content typically below 2.2mmol/L, and reduced chloride levels. Gram staining positivity may exceed 60%, and bacterial culture positivity may exceed 80%.

(c) MRI scans were more diagnostically valuable than CT scans, showing no early abnormalities in the disease course. With disease progression, T1-weighted imaging may reveal subarachnoid space high signal intensity with irregular enhancement, while T2-weighted imaging may show meningeal high signal intensity. In the later stages, diffuse meningeal enhancement and brain edema may be observed.

(d) In other scenarios, pathogenic microorganisms may be detected via blood culture. If petechiae are present, tissue biopsy and bacterial staining should be performed.

③Definitive Diagnosis: Bacterial positivity in cerebrospinal fluid (metagenomic next-generation sequencing).

④Reasonable exclusion of other causes.

### Experimental method

The cerebrospinal fluid sample is collected within 2 days after the patient’s admission and stored in a -80℃ refrigerator.ELISA kit was purchased from Wuhan Cusabio Co. Ltd., China(CSB-E08721h). ELISA assay was performed following the manufacture’s instructions and the lowest sensitivity for detection is 0.039 ng/ml, and the detection range is distributed between 0.156 ng/ml and 20 ng/ml. Briefly, for MPO detection, patients’cerebrospinal fluid was diluted at 1:1, and 100ul diluted cerebrospinal fluid was added to each well for incubation for 2 h. After washing, enzyme-conjugated reagent was added for 60 min. After another round of washing, the substrate for enzyme was added for 60 min. Stop buffer was added and OD value was measured at 450 nm. Standard curve was established using the OD values from controls. The value of tested samples was calculated based on the standard curve. For the detection of CSF-IL-6;CSF-IL-8;CSF-IL-18;CSF-IL-1β;CSF-TNF-α, the procedure is similar to the above operation.

### Clinical outcomes

All patients had accomplished the follow-up of 1 months after discharge. The modified Rankin Scale (mRS) at 1 months after discharge was used to evaluate the clinical outcomes [9, 10]. In our investigation, all participants were stratified into two cohorts: individuals with an mRS score of 0–1, indicative of a good prognosis (*n* = 15); and those with an mRS score of 2–6, denoting an poor prognosis (*n* = 10).

### Statistical analysis

If the measurement data of quantitative data are normally distributed, they are expressed as mean ± standard deviation. If the measurement data are skewed, they are presented as median with IQR (interquartile range). Independent sample t-tests are used for comparisons between two groups, while one-way analysis of variance is used for comparisons among multiple groups. Count data are represented as n (%), and differences between the experimental and control groups are analyzed using the chi-square test. Graphpad Prism 9.0 software is utilized for data visualization. Statistical analysis is conducted using SPSS 26.0 and Graphpad Prism 9.0, with a multivariable logistic regression model evaluating the correlation of CSF-MPO with CSF-IL-1β, CSF-IL-6, CSF-IL-8, CSF-IL-18, and CSF-TNF-α. A p-value of less than 0.05 indicates statistical significance. The optimal cutoff value for prognostic assessment of NETs-related protein content in bacterial meningitis is determined through Receiver Operating Characteristic (ROC) curve analysis.

## Results

### Prognostic differential analysis of neutrophil extracellular trap-associated proteins in patients with bacterial meningitis

We utilized mRS scoring to assess the recovery of patients, with a mRS score of 0–1 indicating a normal range and patient recovery. Patients were followed up for the duration of their recovery prognosis, and the bacterial meningitis patients (*n* = 25) were divided into poor prognosis group (*n* = 10) and good prognosis group (*n* = 15)**(** Table 1**)**. The aim of this study is to compare the prognostic value of neutrophil extracellular trap-related proteins in patients with bacterial meningitis.

**Table 1:** Laboratory examination of the good prognosis group and the poor prognosis group in the group of bacterial meningitis

Project	Poor Prognostic group (*n* = 10) (median ± standard deviation)	Good prognosis group(*n* = 15) (median ± standard deviation)	*P* value
Age	45.40 ± 16.17	40.40 ± 4.64	0.2661
Sex			0.6865
Female%	2(20)	3(20)	
CSF-MPO(ng/ml)	21.68 ± 5.48	14.57 ± 6.28	0.0079
CSF-IL-6(pg/ml)	170.46 ± 54.25	94.44 ± 58.82	0.0034
CSF-IL-8 (pg/ml)	6743.81 ± 2078.98	4001.37 ± 2246.25	0.0053
CSF-IL-18 (pg/ml)	52.95 ± 17.07	32.33 ± 14.09	0.0032
CSF-IL-1β(pg/ml)	5.82 ± 1.91	3.59 ± 1.63	0.0047
CSF-TNF-α(pg/ml)	47.44 ± 13.80	30.35 ± 11.45	0.0026

**Fig. 1:**
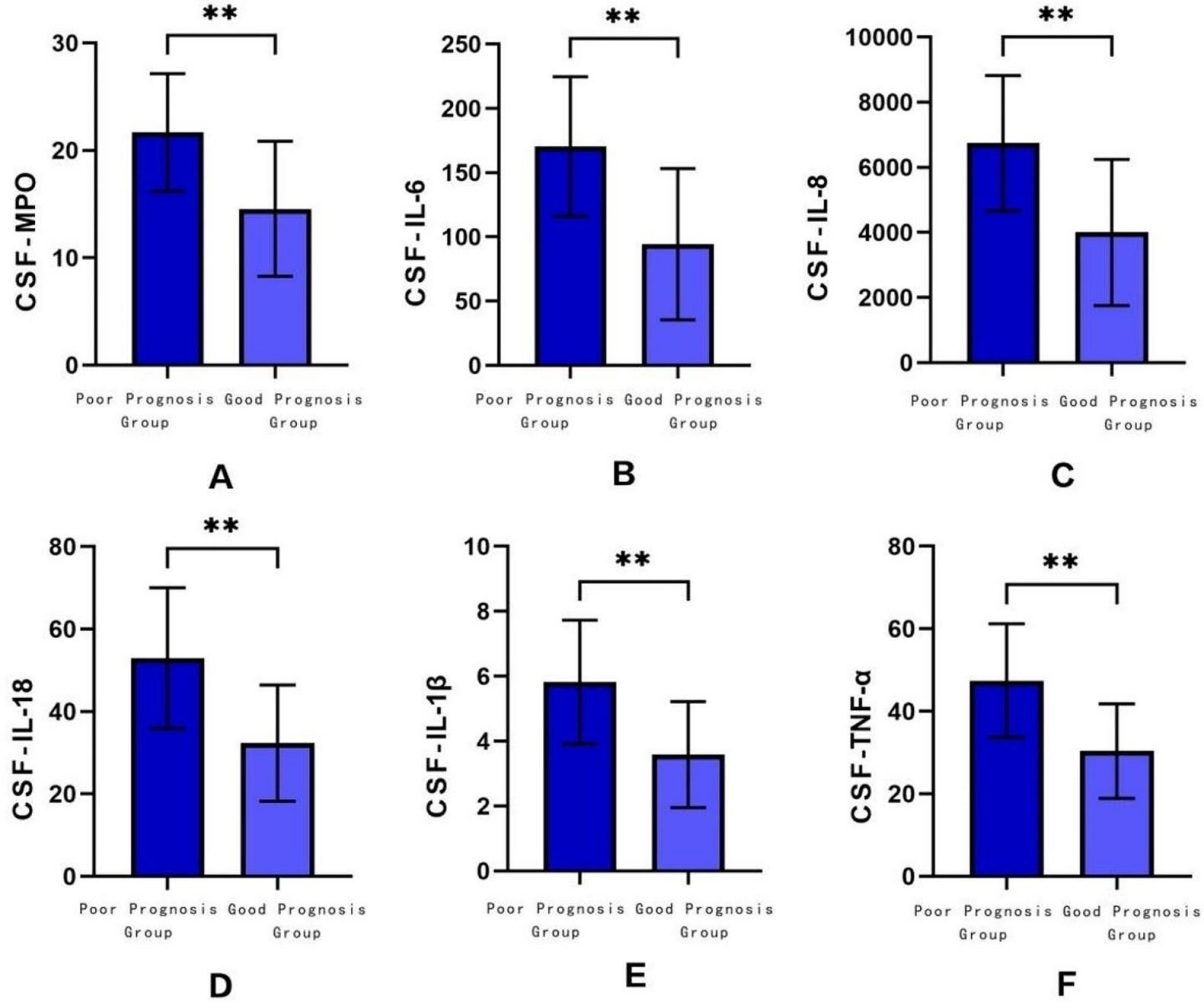
Differential analysis of relevant indicators between poor prognosis group and good prognosis group (**A**) differential analysis of CSF-MPO (**B**) differential analysis of CSF-IL6 (**C**) Differential analysis of CSF-IL8 (**D**) differential analysis of CSF-IL18 (**E**) differential analysis of CSF-IL1β (**F**) differential analysis of CSF-TNFα nsP > 0.05; ****P < 0.0001; ***P < 0.0002; **P < 0.0021; *P < 0.0332

The results of the Shapiro-Wilk test indicated that the data were normally distributed. For the detection indicators required, a T-test was employed. The results revealed (Table 1): There were no significant differences in age or gender among the patients. In terms of laboratory indicators, the statistical description of CSF-MPO, CSF-IL-6, CSF-IL-8, CSF-IL-18, CSF-IL-1β, and CSF-TNF-α in the poor prognosis group of bacterial meningitis were: 21.68 ± 5.48, 170.46 ± 54.25, 6743.81 ± 2078.98, 52.95 ± 17.07, 5.82 ± 1.91, 47.44 ± 13.80, respectively. In the good prognosis group, the statistical descriptions of CSF-MPO, CSF-IL-6, CSF-IL-8, CSF-IL-18, CSF-IL-1β, and CSF-TNF-α were: 14.57 ± 6.28, 94.44 ± 58.82, 4001.37 ± 2246.25, 32.33 ± 14.09, 3.59 ± 1.63, 30.35 ± 11.45, respectively. There was a significant difference between the inflammatory factors in the poor prognosis group and the good prognosis group (*P* < 0.05) (Fig. 1).

### Correlation analysis of neutrophil extracellular trap granule protein MPO and associated inflammatory proteins

**Fig. 2:**
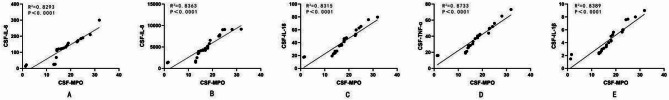
Correlation Analysis between MPO and Related Routine Test Indicators: **A**: MPO has a positive correlation with CSF-IL-6 content (R2 = 0.8293, *P* < 0.0001); **B**: MPO has a positive correlation with CSF-IL-8 (R2 = 0.8363, *P* < 0.0001); **C**: MPO has a positive correlation with CSF-IL-18 (R2 = 0.8315, *P* < 0.0001); **D**: MPO has a positive correlation with CSF-TNF-α (R2 = 0.8733, *P* < 0.0001); **E**: MPO has a positive correlation with CSF-IL-1β (R2 = 0.8389, *P* < 0.0001)

We conducted a correlation analysis between MPO values and clinically significant test indicators to demonstrate that MPO is significantly correlated with inflammatory proteins in bacterial meningitis(Fig. 2). By reviewing literature and analyzing data, we can conclude that the presence of MPO in cerebrospinal fluid is based on the release of neutrophil extracellular traps (NETs) killing granules into the extracellular space. The antimicrobial proteins from neutrophil extracellular traps remain in the body longer than the DNA scaffold [11]. Therefore, MPO can be detected in cerebrospinal fluid, and inflammatory proteins such as IL-6, IL-8, and IL-18 are involved in neutrophil chemotaxis, inducing their entry into the cerebrospinal fluid and promoting the release of NETs. Hence, MPO is correlated with inflammatory proteins.

### Prognostic evaluation value of neutrophil extracellular trap-related proteins

**Table 2:** Sensitivity, specificity, PPV, NPV and AUROC of cerebrospinal fluid neutrophil-related indicators in the diagnosis of bacterial meningitis

Diadynamic criteria	Cutoff	Sensitivity %	Specificity %	PPV (%)	NPV (%)	AUROC (95% CI)	*P*-value
CSF-MPO (ng/ml)	16.57	90	66.7	64.3	95.2	0.83(0.68–0.99)	0.006
CSF-IL-6 (pg/ml)	123.92	90	66.7	64.3	95.2	0.83(0.68–0.99)	0.006
CSF-IL-8 (pg/ml)	4230.62	90	66.7	64.3	95.2	0.83(0.68–0.99)	0.006
CSF-IL-18 (pg/ml)	35.55	90	66.7	64.3	95.2	0.83(0.68–0.99)	0.006
CSF-IL-1β (pg/ml)	3.83	90	66.7	64.3	95.2	0.83(0.68–0.99)	0.006
CSF-TNF-α (pg/ml)	35.19	90	66.7	64.3	95.2	0.84(0.68–0.99)	0.005

**Fig. 3:**
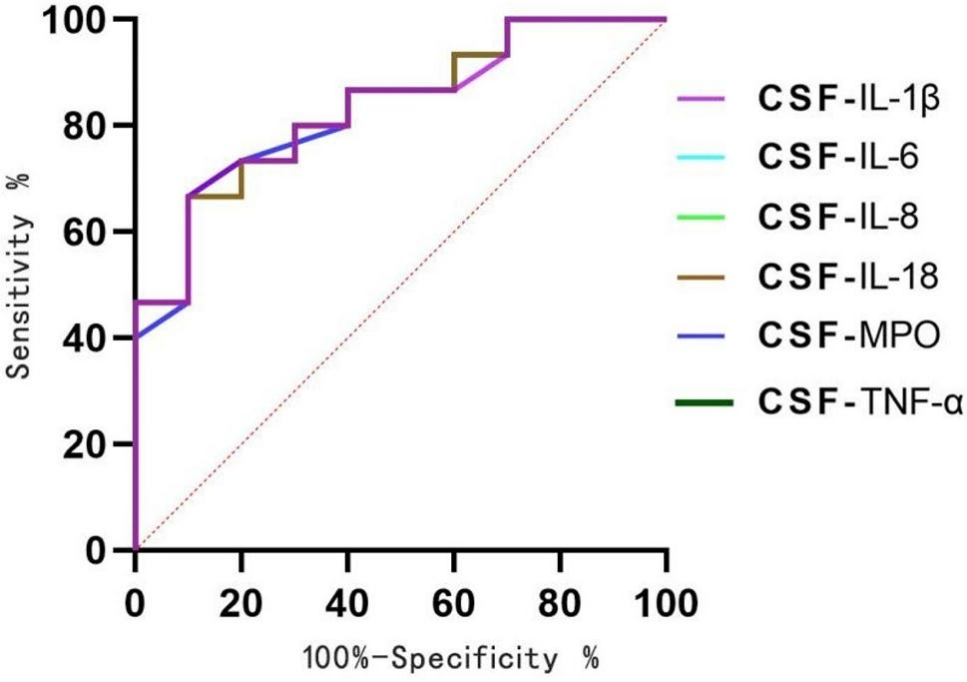
ROC curve. The Receiver Operating Characteristic (ROC) curves for Cerebrospinal Fluid (CSF) levels of Interleukin CSF-IL-1β, CSF-IL-6, CSF-IL-8, CSF-IL-18, CSF-MPO, and Tumor Necrosis Factor CSF-TNF-α at cutoff values of 3.83pg/mL, 123.92pg/mL, 4230.62pg/mL, 35.55pg/mL, 16.57ng/mL, and 35.19pg/mL, respectively

The sensitivity, specificity, positive predictive value (PPV), and negative predictive value (NPV) of cerebrospinal fluid cytokines for predicting the prognosis of bacterial meningitis are shown in (Table 2). For the diagnosis of bacterial meningitis, based on the ROC curve, at the optimal cutoff level of 16.57ng/mL, CSF-MPO demonstrated 90% sensitivity, 66.7% specificity, 64.3% PPV, and 95.2% NPV. At the optimal cutoff level of 123.92pg/mL, CSF-IL-6 showed 90% sensitivity, 66.7% specificity, 64.3% PPV, and 95.2% NPV. Similarly, at the optimal cutoff levels of 4230.62pg/mL, 35.55pg/mL, 3.83pg/mL, and 35.19pg/mL, CSF-IL-8, CSF-IL-18, CSF-IL-1β, and CSF-TNF-α demonstrated 90% sensitivity, 66.7% specificity, 64.3% PPV, and 95.2% NPV.

Based on the above analysis, it can be concluded that neutrophil extracellular trap-related markers have some reference value in predicting the prognosis of bacterial meningitis. When CSF-MPO is ≥ 16.57ng/mL, there is an 83% probability of having a poor prognosis of bacterial meningitis, as well as when CSF-IL-1β, CSF-IL-6, CSF-IL-8, CSF-IL-18, and CSF-TNF-α levels are at 3.83pg/mL, 123.92pg/mL, 4230.62pg/mL, 35.55pg/mL, and 35.19pg/mL respectively, there is an 83% probability of having a poor prognosis of bacterial meningitis(Fig. 3).

Bacterial meningitis is a severe disease caused by bacteria, with symptoms including high fever, severe headache, nausea, vomiting, and stiff neck [12]. Children and adult patients often present with fever, severe headache, vomiting, seizures, altered consciousness, or neck stiffness during the acute phase of the disease. Newborns and infants may have high fever, irritability, sleepiness, breathing difficulties, jaundice, followed by seizures, opisthotonus, and apnea, with rare neurological manifestations. Newborns may have a history of premature birth, birth trauma, or maternal infection before illness. Early signs of meningeal irritation such as neck stiffness, positive Kernig sign, and positive Brudzinski sign may be present during physical examination. However, neck stiffness in infants and young children is often not obvious, and may manifest as bulging fontanelles and opisthotonus. Clinical symptoms are atypical, and it is difficult to distinguish from other infectious diseases in the early stages, therefore, this study analyzes existing literature, combined with current diagnostic protocols, to explore whether there are markers in the cerebrospinal fluid that can be used to predict the prognosis of bacterial meningitis.

Under normal circumstances, neutrophils are kept out of the cerebrospinal fluid due to the presence of the blood-brain barrier. Therefore, mononuclear cells are primarily present in the cerebrospinal fluid. However, in various pathological conditions such as infection, trauma, brain ischemia, neurodegenerative infiltration, or autoimmune conditions, neutrophils can cross this barrier and enter the cerebrospinal fluid [13, 14], providing a theoretical basis for the presence of NETs and related inflammatory factors in the cerebrospinal fluid.

The mechanism of neutrophil killing bacteria by releasing NETs was discovered by the Brinkmann team in 2004. NETs are mesh-like structures released into the extracellular space by activated neutrophils, consisting of a backbone of free deoxyribonucleic acid embedded with proteins such as histones, neutrophil elastase, myeloperoxidase (MPO), and proteinase G [15].

Since the discovery of NETs, they have gradually been applied in the diagnosis of infectious diseases due to their origin from immune cells, such as bacteria [15], viruses [16, 17], and fungi [18, 19]. Research has shown that the formation of NETs can limit the activity of pathogens within a certain range and assist other immune cells in achieving immune defense. However, an excess formation of NETs can worsen the condition, such as accelerating the development of autoimmune diseases and facilitating tumor metastasis. In different diseases, inflammation proteins associated with NETs play a key role. The Cesta MC team found that neutrophils play an important role in the immune response to infection-induced acute respiratory distress syndrome in COVID-19 [20]. They trigger the formation of NETs, leading to the production of cytokines including CSF-IL-8/CSF-CXCL-8, CSF-IL-6, CSF-IL-1β, CSF-TNF-α, and mediate the recruitment of other immune cells to regulate acute and chronic inflammation processes that can lead to ARDS. CXCL8 is involved in the recruitment, activation, and degranulation of neutrophils, thus contributing to the amplification of inflammation and the severity of the disease. However, investigations into NETs-related proteins within the central nervous system remain relatively sparse. This study presents pioneering evidence of the existence of NETs-related proteins in cerebrospinal fluid, showcasing a degree of variability and offering prognostic predictive capabilities for patients.

Currently, there is still a lack of research literature on the clinical diagnosis of NETs. This study validates the clinical diagnostic value of NETs-related proteins in bacterial meningitis and attempts to determine their prognostic reference values, which is considered innovative. The detection method only requires ELISA testing of cerebrospinal fluid, making it convenient for laboratory personnel with low costs. This method is suitable for early hospital admission testing, improving the diagnostic efficiency of bacterial meningitis.

Presently, a dearth of scholarly literature exists concerning the clinical diagnosis of NETs. This study substantiates the clinical diagnostic utility of NETs-related proteins in bacterial meningitis while endeavoring to ascertain their prognostic benchmarks. Notably, the detection methodology merely necessitates ELISA analysis of cerebrospinal fluid, rendering it accessible to laboratory personnel with minimal costs. This approach proves conducive to early hospital admission testing, thereby enhancing the diagnostic efficacy of bacterial meningitis.

However, this experiment does have some limitations. Firstly, as this study is retrospective, it is difficult to control for confounding factors. Secondly, the acquisition of patient cerebrospinal fluid samples depends on patient consent, requiring patient approval for lumbar puncture examination in clinical practice. Therefore, this study is a small-sample single-center study, inevitably carrying a risk of bias. Thirdly, this experiment used metagenomic next-generation sequencing to only select patients with clear pathogen diagnostic criteria, potentially overlooking some undetected patients due to the complexity of clinical situations.

In conclusion, our findings underscore the potential of NETs-related protein levels as clinical markers for bacterial meningitis. This revelation stands poised to aid clinicians in the prompt identification of critically afflicted patients with bacterial meningitis, thereby optimizing clinical management strategies and enhancing patient prognoses to a significant degree.

## Discussion

The emergence of Neutrophil Extracellular Traps (NETs) as a novel facet of neutrophil-mediated immune function has spurred extensive inquiry into their microbial entrapment capabilities. Researchers have delved into their mechanisms, unveiling their multifaceted effects on inflammation, autoimmune disorders, and malignancies. In related investigations, the functional duality of NETs has come to the fore, as their immunomodulatory properties harbor potential, yet their ultimate impact remains enigmatic. Whether NETs confer benefits or inflict harm hinges upon various factors, including activation modalities, dysregulation of inhibitory mechanisms, and NETs’ abundance, all of which could wield pivotal roles in disease pathogenesis. Thus, the quantification of NETs assumes paramount importance.

Given the heightened stability of NETs-associated proteins in cerebrospinal fluid, our inquiry into the influence of NETs on disease outcomes through these proteins bears substantial clinical relevance. Nonetheless, myriad aspects await exploration by future researchers. Leveraging the strides made in modern diagnostic modalities, our endeavor in scrutinizing NETs aims to deepen our comprehension of their functionality and immunological repercussions, with the overarching objective of attenuating and ultimately obliterating their deleterious effects while preserving their beneficial contributions.

## Data Availability

The original contributions presented in the study are included in the article, further inquiries can be directed to the corresponding author：wang_yun@email.sdu.edu.cn.
